# Differential Analysis of Three Copper-Based Nanomaterials with Different Morphologies to Suppress *Alternaria alternata* and Safety Evaluation

**DOI:** 10.3390/ijms24119673

**Published:** 2023-06-02

**Authors:** Zitong Yuan, Yiwei Li, Yuke He, Kun Qian, Yongqiang Zhang

**Affiliations:** College of Plant Protection, Southwest University, Chongqing 400715, China; yuanzitongt@163.com (Z.Y.); lywwei599@163.com (Y.L.); hyk0002022@163.com (Y.H.)

**Keywords:** cuprous oxide nanoparticles, copper nanorods, copper nanowires, nano-pesticide, zebrafish, *Alternaria alternata*

## Abstract

The overuse of copper-based fertilizers and pesticides over the last few decades has resulted in detrimental risks to our environment. Nano-enabled agrichemicals with a high effective utilization ratio have shown great potential for maintaining or minimizing environmental issues in agriculture. Copper-based nanomaterials (Cu-based NMs) serve as a promising alternative to fungicides. Three types of Cu-based NMs with different morphologies were analyzed for their different antifungal effects on *Alternaria alternata* in this current study. Compared to commercial copper hydroxide water power (Cu(OH)_2_ WP), all tested Cu-based NMs, including cuprous oxide nanoparticles (Cu_2_O NPs), copper nanorods (Cu NRs) and copper nanowires (Cu NWs), especially Cu_2_O NPs and Cu NWs, showed higher antifungal activity against *Alternaria alternata*. Its EC_50_ were 104.24 and 89.40 mg L^−1^, respectively, achieving comparable activity using a dose approximately 1.6 and 1.9-fold lower. Cu-based NMs could introduce the downregulation of melanin production and soluble protein content. In contrast to trends in antifungal activity, Cu_2_O NPs showed the strongest power in regulating melanin production and protein content and similarly exhibited the highest acute toxicity to adult zebrafish compared to other Cu-based NMs. These results demonstrate that Cu-based NMs could offer great potential in plant disease management strategies.

## 1. Introduction

Copper (Cu) is an essential micronutrient for plant growth, which plays a critical role in various physiological processes [1]. Deficient Cu availability is detrimental to plant growth and development, resulting in lower crop yields [2]. Cu has been applied as an effective pesticide against plant pathogens in agriculture and food preservation [3,4]. Cu-based fertilizers and fungicides have been overused to ensure crop yield and quality. However, there are important concerns regarding their intensive use due to soil contamination, adverse effects on soil fauna, soil microbiomes, phytotoxicity, and metal residues in food [5,6]. The long-term use of Cu-based fungicides has resulted in an excessive accumulation of Cu in the soils of French and Portuguese vineyards [7,8]. In temperate zones, it has been shown that earthworms are more susceptible to Cu than most other groups of soil invertebrates [9]. The excessive accumulation and leaching of Cu from agricultural soils can contaminate groundwater and pose a risk to terrestrial organisms, fish and even human health [10,11,12]. In response to this alert, the European Community has recently lowered the annual maximum Cu limit from 6 kg ha^−1^ to 4 kg ha^−1^ [13,14]. However, Cu-based fungicides are still an irreplaceable strategy for planning disease control. Therefore, it is urgent to develop new Cu-based fungicides with low doses and high antifungal activity to alternate traditional copper fungicides. Nano-enabled agrichemicals have attracted research attention as nanotechnology is seen as deeply essential for reducing severe environmental problems from current agriculture. A series of Cu-based nanomaterials (NMs) have been introduced into agriculture application research, including Cu, Cu(I) and Cu(II)-based NMs. It has been documented that antimicrobial activity is based on adhesion, oxidative stress and the dissolution of Cu(I) and Cu(II) ions [15]; these ions can damage the cell wall and plasma membrane and result in cell death [16,17]. Cu(II)-based nanofibers have a synergistic antibacterial mechanism, which is based on the generation of reactive oxygen species (ROS) and the dissolution of Cu(II) ions [18]. The Cu(I) state is shown to possess higher antimicrobial potency than the Cu(II) state, and the cycling between Cu(I) and Cu(II) generates a superoxide species, which causes oxidative stress via ROS [19,20]. The discrepancy in the antibacterial activity of Cu-based NMs with different morphologies and oxidation states has aroused extensive research in relation to their size effect and dissolution behaviors. Zinc oxide has shape-dependent antimicrobial activity against *E. coli* and *P. aeruginosa* [21]. Compared to copper oxide nanoparticles (CuO NPs), Cu-based nanosheets have shown stronger disease suppression due to their greater leaf surface affinity and quicker Cu(II) ion dissolution [22]. Despite these reports, the exact differences in the antibacterial ability and synergistic mechanisms of Cu-based NMs require further clarification.

*Alternaria alternata* (*A. alternata*) is a worldwide extremely destructive plant pathogen that infects more than 100 plant species, causes a variety of black spot diseases [23,24], and results in the decline of food production and quality, which contributes to tremendous economic losses. It is also a common post-harvest fungus and causes food rot and contamination [25,26]. Diverse phytotoxin production is responsible for pathogenicity [27]. *A. alternata* produces melanin during growth, which protects the fungus against environmental stresses, such as metal ions [28,29]. Copper ions stimulate melanin production in the fungi *Paecilomyces variotii* and *Aspergillus carbonarius* [30]. However, the interaction of melanin with metal-based compounds and the specific regulation of its virulence and pathogenesis needs to be investigated further.

Due to their outstanding antifungal activity, Cu-based NMs are gaining popularity. CuO NPs, via biological synthesis, can achieve an antifungal effect by influencing the sporulation pattern of *Alternaria brassicae* [31]. Column-shaped CuO NPs (50 nm in diameter and 70–100 nm in length) effectively suppress *A. alternata* at a low dosage (10 mg L^−1^) [32]. However, the toxicity of Cu to fish cannot be ignored. Therefore, it is necessary to proceed through a strict safety evaluation before a new Cu-based fungicide can be developed and used in the field. Numerous studies have reported the toxic behavior of Cu-based NMs in aquatic systems. For instance, the toxicity of Cu compounds is commonly ranked by high throughput assays, such as Cu(II) > nano Cu > micron-scale Cu compounds, due to Cu NPs, which can damage DNA plasmids through the generation of ROS [33]. Both Cu NPs and releasing Cu(II) ions can mediate their toxicity effects on zebrafish embryos in a dose-dependent manner [34]. However, the Cu ion release in the extracellular environment of Cu-based NMs does not completely explain this toxicity difference. Transformation and intracellular processes in Cu-based NMs may help explain their toxic behavior [35].

The U.S. Environmental Protection Agency has approved elemental Cu and Cu-based compounds as antimicrobials [36]. Previous studies have reported the antifungal activity of a wide range of novel Cu-based compounds, but few comparative studies have focused on the differences in toxicity of different Cu-based fungicides. In this paper, homogeneous cuprous oxide NPs (Cu_2_O NPs) were reported via a more convenient method. Antifungal activities and the down-regulation of soluble protein contents were investigated with three types of Cu-based NMs, Cu_2_O NPs, copper nanorods (Cu NRs) and copper nanowires (Cu NWs). Due to the non-negligible toxicity behavior of Cu, the acute toxicity of Cu-based NMs was evaluated on zebrafish as a model aquatic organism. Based on safety assessment, this study focused on the different antifungal activities of three Cu-based NMs with different morphologies to provide a new management strategy for plant pathogen control with Cu-based fungicides.

## 2. Results and Discussion

### 2.1. Cu-Based NMs Characterization

**Figure 1:**
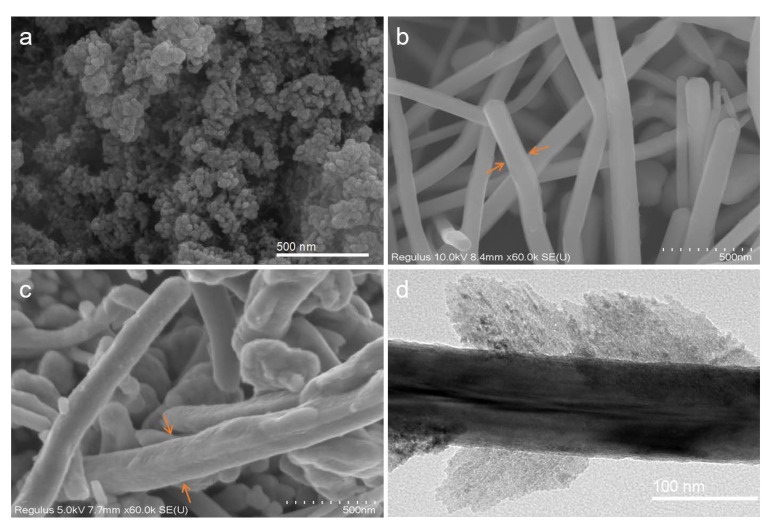
SEM images of (**a**) Cu_2_O NPs, (**b**) Cu NRs and (**c**) Cu NWs. (**d**) TEM image of Cu NRs.

**Figure 2:**
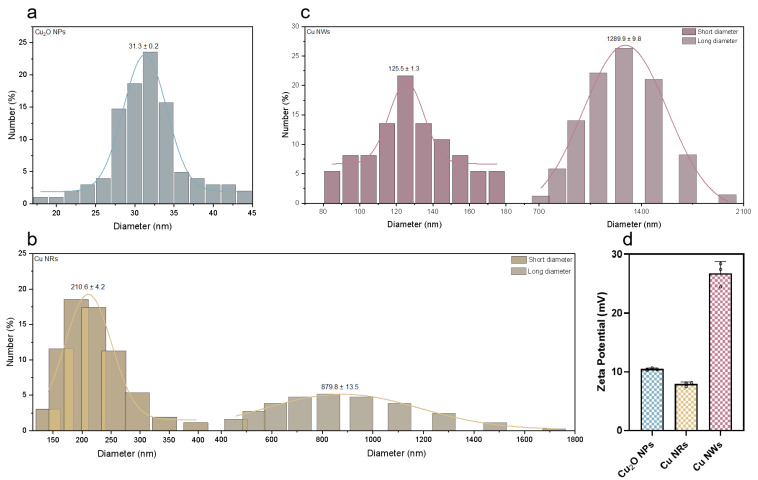
The size distribution analyses of (**a**) Cu_2_O NPs, (**b**) Cu NRs and (**c**) Cu NWs. (**d**) *Zeta* potential of Cu_2_O NPs, Cu NRs and Cu NWs.

SEM images show that Cu_2_O NPs (Figure 1a and Figure 2a) possess uniform spheroid particles and some degree of agglomeration, with an average diameter of 31.3 nm. Cu NWs (Figure 1b and Figure 2c) adopt a wire-like morphology with an average diameter of 125.5 nm and a length of several microns, the surface of which is smooth (shown in Figure 1b orange arrow). The morphologies of Cu NRs and Cu NWs are similar. The NRs (Figure 1c and Figure 2b) are shorter and thicker than NWs, with an average diameter of 210.6 nm and an average length of 879.8 nm. Moreover, the surface of Cu NRs is covered with a rough oxide layer, which enlarges the surface area (shown in Figure 1c orange arrow). The relatively rough surface of Cu NRs can be clearly observed through the TEM image (Figure 1d). The *zeta* potential of Cu_2_O NPs, Cu NRs and Cu NWs (100 mg L^−1^) in water was +10.4, +7.9 and +26.8 mV, respectively (Figure 2d and Figure S4).

**Figure 3:**
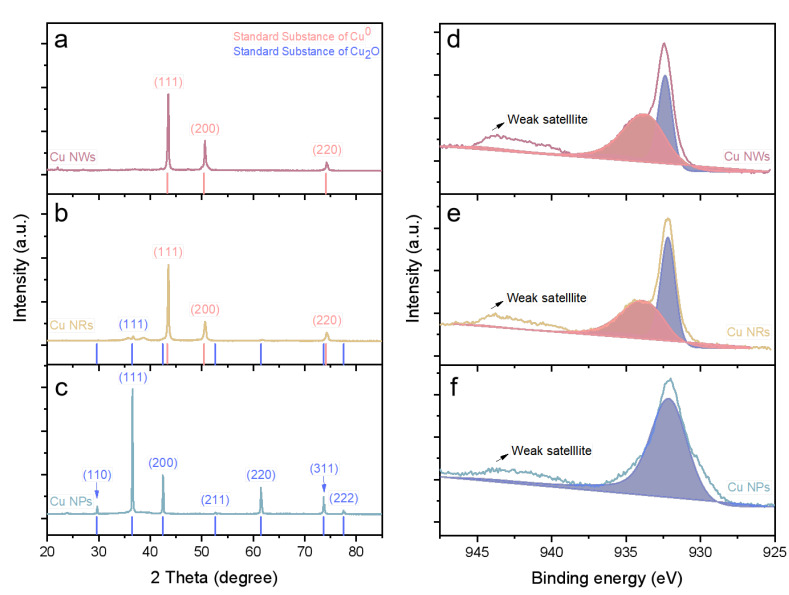
(**a**–**c**) The XRD patterns of Cu_2_O NPs, Cu NRs and Cu NWs. (**d**–**f**) Cu 2p_3/2_ XPS spectra of Cu_2_O NPs, Cu NRs and Cu NWs.

Figure 3a–c shows the XRD pattern of NMs. The Cu_2_O NPs diffraction pattern was consistent with that found by the standard Cu_2_O (JCPDS No. 65-3288). Cu NRs and NWs have three distinctly distinguishable diffraction peaks at 2*θ* of 43.5°, 50.6° and 74.3°, which corresponded excellently to the (111), (200) and (220) crystal planes of Cu (JCPDS NO. 4-836). Both the prepared NPs and NWs had high purity, and there was no peak in any contaminant. By contrast, NRs appeared with an extra peak at 2*θ* of 36.6° corresponding to the (111) reflection of Cu_2_O, which could explain the substance composition of NRs’ rough surface.

Figure 3d,e shows the Cu 2p_3/2_ XPS spectra of Cu-based NMs. A peak of NPs, NRs and NWs was observed at 932.4 eV, with a weak satellite at 943.8 eV. It was difficult to distinguish the peak positions between Cu^0^ and Cu^1+^ because of the weak binding energy difference between these two bonds [37]. However, a basic distinction could be made between peak width and satellite peak. Unlike NPs, which were mainly composed of Cu_2_O, the surface of NRs and NWs had partial Cu_2_O. Another lower peak at 933.8 eV of NRs and NWs can be seen, which could be attributed to the peak position of Cu^2+^.

### 2.2. Antifungal and Soluble Protein Assay

**Figure 4:**
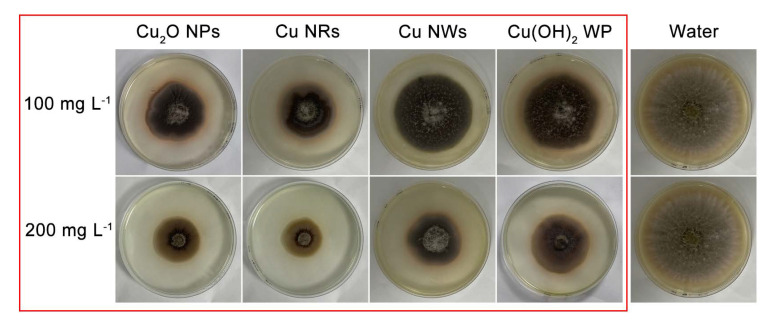
A picture of *A. alternate* colony under blackness cultivation for 7 days with 100 and 200 mg L^−1^ of Cu_2_O NPs, Cu NRs, Cu NWs and Cu(OH)_2_ WP.

Phenotypic images of *A. alternata* upon exposure to NMs and WP showed a good dose–response relationship between the inhibition rate of hyphae (Figure 4 and Figure S1). Colonies exposed to NMs and WP were darker than those treated with sterile water, and red halos could be seen at the edges of the colony, suggesting that oxidative damage or copper ion stress might promote melanin production. Melanin in fungi is often considered a defense mechanism against several environmental stresses, including heavy metals [38,39]. It also contributes to pathogenesis. Colonies respond to stress from Cu NMs by regulating melanin production, but the amount of melanin seems not to be significantly correlated with the dose of copper. However, the effect of melanin on virulence in *A. alternata* requires additional study.

**Figure 5:**
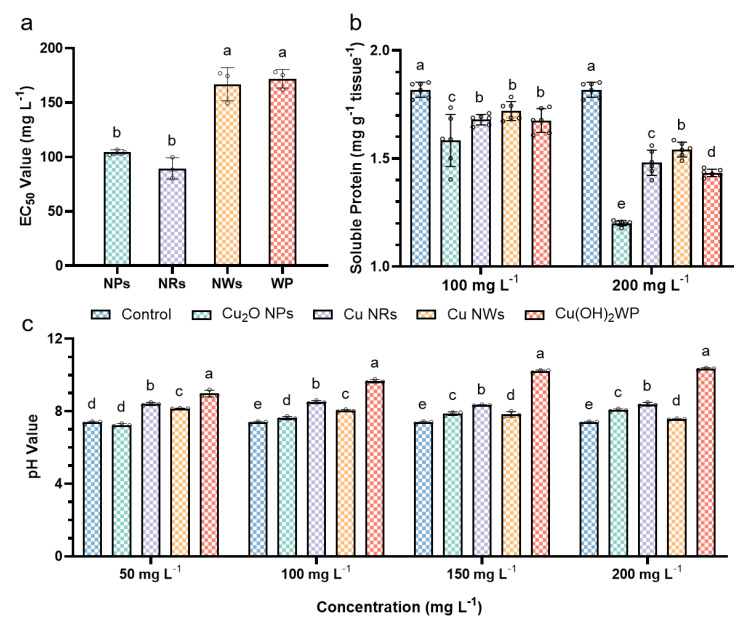
(**a**) The inhibition rate of Cu_2_O NPs, Cu NRs, Cu NWs and Cu(OH)_2_ WP on hyphae of *A. alternate*. (**b**) The soluble protein of *A. alternate* colony 100 and 200 mg L^−1^ of Cu_2_O NPs, Cu NRs, Cu NWs and Cu(OH)_2_ WP treatments. (**c**) The pH values of Cu_2_O NPs, Cu NRs, Cu NWs and Cu(OH)_2_ WP aqueous solutions (50–200 mg L^−1^). Values are means ± SD, *n* = 3 (**a**,**c**), *n* = 5 (**b**). Values shown by different letters were significantly different at *p* < 0.05. One-way ANOVA with *L*SD test used for comparison.

The antifungal activity of NMs was evaluated against *A. alternata* by determining their EC_50_. (Table S3 and Figure 5a). The EC_50_ values of Cu_2_O NPs, Cu NRs, Cu NWs and Cu(OH)_2_ WP against *A. alternata* were 104.24, 89.40, 166.88 and 171.87 mg L^−1^, respectively. Cu NWs had the same antifungal activity as WP, while NPs and NRs had the same antifungal activity but were stronger than WP. Controlling the size and shape of nanopesticides is crucial to achieving an appropriate antibacterial action under physiological conditions [40], and Cu-based NMs have a strong antifungal activity rendered by Cu(II) ions dissolution and oxidative stress [15,41]. As the morphology of Cu-based NMs can directly determine dissolution and accumulation [22], this primarily influences virulence. Cu(I) ions have been shown to be considerably more toxic than Cu(II) ions due to their higher cytoplasmic membrane permeability [42], which is consistent with the results of Cu_2_O NPs, which had high antifungal activity against *A. alternata*. Previous studies have shown that spherically shaped Cu_2_O, Cu/Cu_2_O and CuO NPs of a similar size (11–14 nm) were tested in the field against the late blight of tomato caused by *Phytophthora infestans*. Compared to CuO NPs, Cu_2_O NPs were found to be more effective 3 and 10 days after application, and Cu/Cu_2_O NPs performed more at a more stable level among all days of the assessment compared to CuO and Cu_2_O NPs [36,43]. This is consistent with the results: CuNRs partially coated with Cu_2_O and CuO had higher activity.

The soluble protein content of mycelium (Figure 5b) treated by Cu-based NMs and Cu(OH)_2_ WP decreased significantly, which was obviously different based on dose. At high doses (200 mg L^−1^), the soluble protein content expressed overt differences based on the Cu-based type; Cu_2_O NPs, Cu NRs, Cu NWs and Cu(OH)_2_ WP, respectively, reduced the soluble protein content by 34.07, 18.68, 15.38 and 21.43%, but no such phenomenon was found at low doses (100 mg L^−1^). Cu_2_O NPs had the greatest influence on the content, which further proved that Cu(I) ions had more influence on cytoplasmic membrane permeability than Cu(II) ions. However, it seemed that Cu-based NM’s down-regulation of the protein content by Cu-based NMs was not related to their virulence, which was probably due to the different dissolution rates of Cu(I) or Cu(II) ions in Cu-based NMs.

### 2.3. pH Values for Cu-Based NMs

Figure 5c shows the pH values for Cu-based NMs and WP aqueous solutions at different concentrations. All Cu-based NMs and Cu(OH)_2_ WP had significantly higher pH values than the control except for the 50 mg L^−1^ NPs solution. The pH of Cu_2_O NPs and Cu(OH)_2_ WP increased with increasing doses, while the pH of Cu NRs showed no overt relationship with the concentration. Due to their physical properties, Cu NWs decreased in pH with increasing concentration and were more likely to aggregate at high concentrations. The aqueous solution of Cu(OH)_2_ WP was highly alkaline (8.9–10.3), whereas the aqueous solution of Cu_2_O NPs, Cu NRs and Cu NWs was all weakly alkaline (7.2–8.5). Previous studies have shown that pH values above 5.5 lead to a reduction in mycotoxin production [44] in *A. alternata*. Therefore, Cu-based NMs might have great potential as fungicides to protect agricultural products from mycotoxin contamination.

### 2.4. Safety Evaluation of Adult Zebrafish

Zebrafish is a biological model for a chemical toxicity assessment [45]. The behavioral responses of fish in Cu-based NMs were similar. All tested fish showed a sudden jump in the first 30 min, followed by toxic phenomena such as reduced activity and slow response, and finally, the death of the fish showed gill hyperemia (Figure S2). The 96 h LC_50_ value of Cu_2_O NPs, Cu NRs and Cu NWs to the zebrafish was 0.52, 1.31 and 5.27 mg L^−1^, respectively (Table 1). High pH levels (9–14) can harm fish by denaturing cellular membranes [46]; the dispersion of Cu-based NMs caused pH changes (less than 1) in the water body, which could be ignored by fish adaptation. For the Cu NWs group, reddish-brown suspended solids were seen around the gills of dead fish, whose trace amounts of Cu could induce the aggregation and sedimentation of Cu NWs in the water. This decreased the amount of Cu NWs in the water, which might be responsible for the lowest toxicity of Cu NWs. Compared to other Cu-based NMs, Cu_2_O NPs have the unique tendency to alternate between both Cu(I) and Cu(II) oxidation states [47]. Furthermore, switching between these states generates oxygen species and induces cell death [48,49], which might explain the highest acute toxicity of Cu_2_O NPs in zebrafish. Until now, the mechanisms responsible for the different toxic behavior of Cu-based NMs based on their types remain largely unknown and need further investigation.

## 3. Materials and Methods

### 3.1. Chemicals and Instruments

Unless otherwise indicated, all reagents (analytical grade) were acquired from Aladdin (Shanghai, China) and were used without additional purification. The experimental water was prepared by an ultrapure system (18.25 MΩ cm^−1^, Human Power I+, Beijing General Analytical Corporation, Beijing, China). *A. alternata* was provided by Prof. Jia’s laboratory at the Guizhou Academy of Tobacco Science in China.

### 3.2. Preparation of Cu-Based NMs

Cu_2_O NPs were prepared by liquid-phase reduction. In total, 2.5 mL of cupric nitrate (Cu(NO_3_)_2_·3H_2_O, 0.5 mol L^−1^, CAS No: 10031-43-3) was added to sodium hydroxide (NaOH, CAS No: 1310-73-2) solution (50 mL, 15 mol L^−1^) with an ultrasonic process. Later, this solution was stable dropwise mixed with 10 mL sodium borohydride (NaBH_4_, 1 mol L^−1^, CAS No: 16940-66-2) with constant stirring for 30 min, and the formation of Cu_2_O NPs was marked by the development of a characteristic dark red solution. The solution was centrifuged at 10,000 r min^−1^ to obtain a precipitate, which was washed twice alternately with deoxygenated water and anhydrous ethanol. It was kept at room temperature after freeze-drying (SCIENTZ-12N, Chongqing, China).

Cu NRs were prepared by the method reported with slight modification by Liu et al. [50]. In brief, 2 mL of Cu(NO_3_)_2_·3H_2_O (0.1 mol L^−1^), 300 µL of ethylene diamine (EDA, CAS No: 107-15-3), and 22 µL of hydrazine hydrate (CAS No: 7803-57-8) were successively added to 20 mL NaOH (15 mol L^−1^) with thorough stirring at 400 r min^−1^. Then, the solution was transferred into an organic glass bottle and incubated for 60 min in a 110 °C oven. The formation of Cu NRs was marked by a change from a dark blue solution to a fluffy wine-red-colored substance, which was suspended above the transparent solution. Subsequently, the solution was cooled to room temperature and centrifuged at 10,000 r min^−1^ to obtain a precipitate, which was washed three times in deoxygenated water. It was kept at room temperature after freeze-drying.

Cu NWs were prepared following the approach described in the previous literature with modifications [51]. Copper chloride (CuCl_2_·2H_2_O, 12.5 mmol L^−1^, CAS No: 10125-13-0) and glucose (C_6_H_12_O_6_·H_2_O, 18.75 mmol L^−1^, CAS No: 5996-10-1) were dissolved in 80 mL of ultrapure water. Then, hexadecylamine (37.5 mmol L^−1^, CAS No: 143-27-1) was slowly added to the solution, followed by vigorous mixing until a light blue emulsion was obtained. The emulsion was transferred into a polytetrafluoroethylene-lined autoclave of a 100 mL capacity and incubated for 180 min at a 120 °C oven. The formation of Cu NWs was marked by a reddish-brown solution, centrifuged at 10,000 r min^−1^, and washed several times alternately with deoxygenated water, *n*-hexane and anhydrous ethanol. This product was kept at room temperature after freeze-drying.

### 3.3. Characterization of Cu-Based NMs

The morphology of Cu-based NMs was assessed by scanning electron microscope (SEM; Hitachi SU8100, Shenzhen, China) and transmission electron microscopy (TEM; JEM2001f, Zhangzhou, China ). The Malvern ZEV3600 (Chongqing, China) was used to measure the size distribution and *Zeta* potential of Cu_2_O NPs, Cu NRs and Cu NWs. Pwder X-ray diffraction (XRD) patterns (Cu K*α*) were recorded by Rigaku Smartlab SE (Shenzhen, China) and scanned at a step of 1° (2*θ*) in a range from 20° to 80°. X-Ray photoelectron spectroscopy (XPS) measurements were conducted by an Thermo Fisher Scientific K-Alpha (Shenzhen, China) instrument with Al K*α* radiation.

### 3.4. A. alternata Culture

To study the effect of Cu NMs on *A. alternata*, the *A. alternata* cake was connected to the culture medium of potato dextrose agar (PDA) plate (46 g L^−1^) and transferred to a biochemical incubator (DGL-30; DENGGUAN Medical Corporation, Chongqing, China). The plate was incubated at 28 ± 1 °C in the dark to the plate edge for the subsequent determination of antifungal activity.

### 3.5. Inhibitory Effects of Cu NMs on A. alternata In Vitro

The antifungal activity of Cu NM was assessed in Petri dishes (9 cm in diameter) by agar dilution method [52]. Three types of Cu NM and 46% copper hydroxide (WP) were prepared as the mother liquor with different concentrations (Table S1) using sterile water. Mother liquors and sterile water were added to the PDA medium, which was cooled to about 60 °C after sterilization (the total amount of liquid added to the medium was 1 mL) and poured into sterilized Petri dishes to obtain the final concentration, as shown in Table S1. A 0.7 cm diameter hyphal round cake was inoculated in the plate center and was cultured at 28 ± 1 °C in the dark for 7 days.

The growth inhibition rate was estimated as follows:

$$\mathrm{Growth}\;\mathrm{inhibition}\;\mathrm{rate}=\frac{\mathrm{Colony}\;\mathrm{diameter}\;\mathrm{of}\;\mathrm{control}\;\mathrm{group}-\mathrm{Colony}\;\mathrm{diameter}\;\mathrm{of}\;\mathrm{treantment}\;\mathrm{group}\;}{\mathrm{Colony}\;\mathrm{diameter}\;\mathrm{of}\;\mathrm{control}\;\mathrm{group}\;-0.7\;\mathrm{cm}}\times 100\%$$

The effective control of 50% growth inhibition (EC_50_) was calculated by employing IBM SPSS Statistics 26.0 software.

### 3.6. Determination of Soluble Protein in Mycelia

Commensurate inoculum sizes were cultured into a 40 mL potato dextrose broth medium (26 g L^−1^, sterile) in a shaker (28 ± 1 °C, 180 r min^−1^, dark) for 2 days. Later, 10 mL of three types of Cu NMs and 46% copper hydroxide (WP) was added to obtain the final concentration of 100 and 200 mg L^−1^, respectively. After 4 days of culture, the treatment mycelium was rinsed twice with sterile water and freeze-dried.

The content of soluble protein in mycelia was determined by the Bradford method [53] with bovine serum albumin (BSA) as the standard; a 50 µL crude aqueous extraction and 200 µL Coomassie Brilliant Blue G-250 solution (RHAWN, Shanghai, China) were added into a 96-well microplate with mild vibration, and an optical density (OD) value at 595 nm was recorded by a multi-function microporous plate reader (Molecular Devices SpectraMax iD3) after holding at 25 °C for 5 min. The measurements were performed in triplicate. For the extraction of mycelia, 4 mL Tris-HCl (25 mmol L^−1^) was added to a 200 mg freeze-dried mycelia. After full grinding, the crude aqueous extraction was obtained by centrifugation (4 °C, 10,000 r min^−1^, 10 min).

### 3.7. pH Measurements

The pH values were measured in the ultrapure aqueous solution of Cu NMs and WP at concentrations of 50, 100, 150 and 200 mg L^−1^ using a potentiometer (PHS-3E; INESA instrument, Chongqing, China). Each measurement was repeated in triplicate.

### 3.8. Safety Evaluation of Adult Zebrafish

Zebrafish (*Danio rerio*) adults of the wild-type AB strain were purchased from Experimental Zebrafish Model Center (Wuhan, China) and were incubated in the same batch. The average body length of adult fish (3 months old) was 3.0 ± 0.2 cm, and their weight was 300 ± 100 mg, with half male and half female. The adult acute toxicity assay was conducted according to the OECD standard protocol [54]. The adult fish were exposed to a certain concentration of Cu NM (shown in Table S2) for 96 h; each beaker contained 1 L test solution (using dechlorinated tap water) and 10 fish. Three replicates were set up for each treatment. The mortality of adults was counted every 24 h, and the dead zebrafish were removed in time. Median lethal concentration (LC_50_) was calculated by employing IBM SPSS Statistics 26.0 software.

### 3.9. Statistical Analysis

Statistical software IBM SPSS Statistics 26.0 was used to analyze the results and Student’s *t*-test and One-way ANOVA were used to analyze the results. Origin 2019b was used to fit the models and for science mapping. GraphPad Prism (version 8.0.2) was used for science mapping.

## 4. Conclusions

Sustainable agriculture is one of the most important trends for future agricultural development. In this context, low-dose and high-efficiency alternatives to conventional chemical pesticides are critical. In this paper, we described the morphology, crystal structure and copper valence analysis of the three Cu NMs in detail, with oxidized Cu more or less present on the surface of Cu NRs and Cu NWs. According to the Cabrera–Mott model [55], the formation of Cu(I) or Cu(II) oxide on the surface of Cu-based NMs could not be avoided in the preparation process, which undoubtedly increased the difficulty of analyzing the differences in antibacterial activity and toxic behavior of Cu-based NMs. Compared to commercial Cu(OH)_2_ WP, all tested Cu-based NMs, especially Cu_2_O NPs and Cu NRs, showed greater antifungal activity against *A. alternata*, and EC_50_ values were almost 1.6 and 1.9-fold lower, respectively. Cu-based NMs might introduce melanin production and the downregulation of the soluble protein content. In contrast to trends of antifungal activity, Cu_2_O NPs showed a stronger power when regulating melanin production and protein content. Here, we thought the dissolution of Cu(I) ions played a major role due to their higher cytoplasmic membrane permeability. However, this does not fully explain the difference in antifungal activity. Similarly, Cu_2_O NPs also showed higher acute toxicity in adult zebrafish than other Cu-based NMs. Based on the safety assessment results, it appears that Cu_2_O NPs are not ideal fungicides to control plant pathogens. Differing from Cu(OH)_2_ WP aqueous solutions, Cu_2_O NPs, Cu NRs and Cu NWs aqueous solutions (50–200 mg L^−1^) were all weakly alkaline, which is more conducive to plant adaptation. Overall, this work further demonstrated the feasibility of Cu-based NMs to replace Cu-based fungicides as novel plant disease control and management strategies. However, the internal mechanisms responsible for the differences in antifungal activity of Cu-based NMs with different morphologies remain to be further explored. Although Cu-based nanopesticides are gaining popularity due to their outstanding antifungal activities, extensive research is warranted on their environmental fate, bioavailability, phytotoxicity, plant mineral nutrient content, and toxicity to non-target organisms [36,56].

## Figures and Tables

**Table 1:** Acute toxicity of Cu NMs and Copper Hydroxide (WP) in adult zebrafish.

Target	Regression Equation	LC_50_ (mg L^−1^)	95% Confidence Limit (mg L^−1^)	R^2^
Cu_2_O NPs	y = 1.26 + 4.26x	0.52	0.40–0.65	0.949
Cu NRs	y = −0.75 + 6.84x	1.31	1.15–1.48	0.951
Cu NWs	y = −2.71 + 3.88x	5.27	3.56–7.33	0.903

## Data Availability

The data presented in this study are available upon request from the corresponding author.

## Supplementary Materials

The following supporting information can be downloaded at: https://www.mdpi.com/article/10.3390/ijms24119673/s1.
