# Supplementary material for: Differential Analysis of Three Copper-Based Nanomaterials with Different Morphologies to Suppress Alternaria alternata and Safety Evaluation
Source: Int J Mol Sci. 2023 Jun 2;24(11):9673. doi: 10.3390/ijms24119673 (PMC10253677; doi:10.3390/ijms24119673)
Supplement: Supplementary file 1 [file ijms-24-09673-s001.zip › ijms-2383667-supplementary.pdf]

Differential analysis of three copper-based nanomaterials with different morphologies to suppress *Alternaria alternata* and safety evaluation

Zitong Yuan, Yiwei Li, Yuke He, Kun Qian * and Yongqiang Zhang *

College of Plant Protection, Southwest University, Chongqing 400715, China; yuanzitongt@163.com (Z.Y.); lywwei599@163.com (Y.L.); hyk0002022@163.com (Y.H.)
* Correspondence: qiankun1982@163.com (K.Q.); zyqiang@swu.edu.cn (Y.Z.)

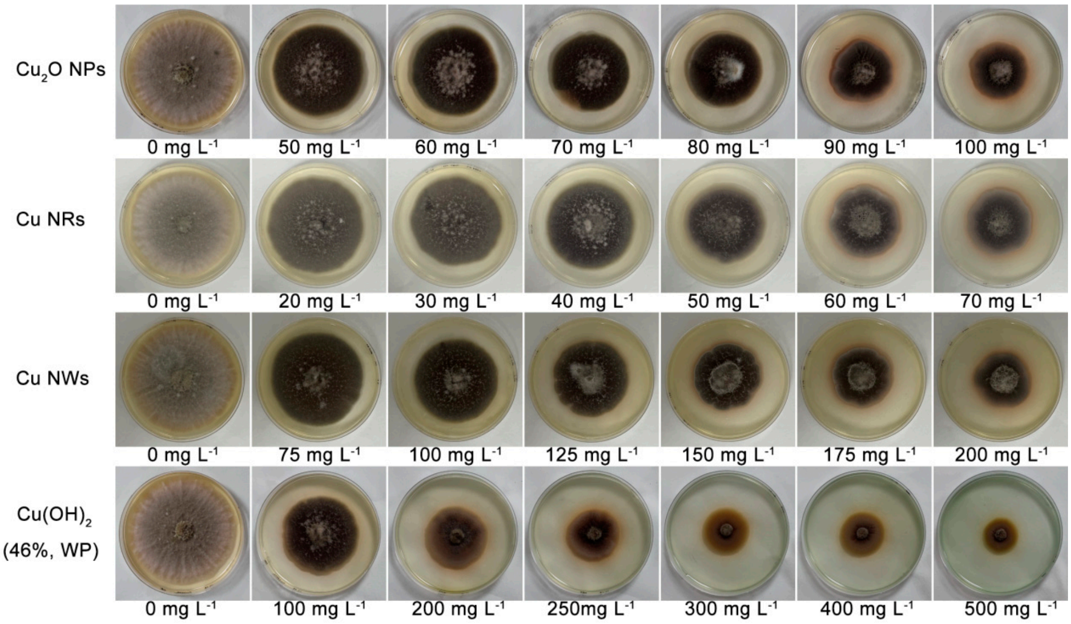

Figure S1. The picture of *A. alternate* colony under blackness cultivation for 7 days with the different concentration of Cu_2O NPs, Cu NRs, Cu NWs and $Cu(OH)_2$ WP.

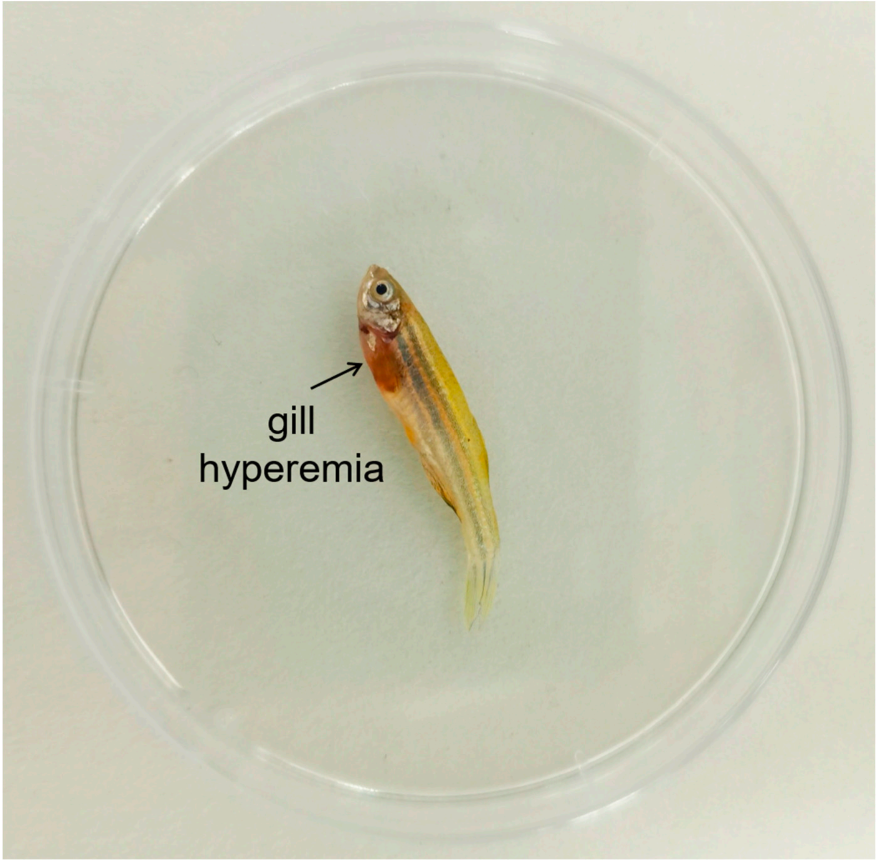

Figure S2. Dead adult zebrafish.

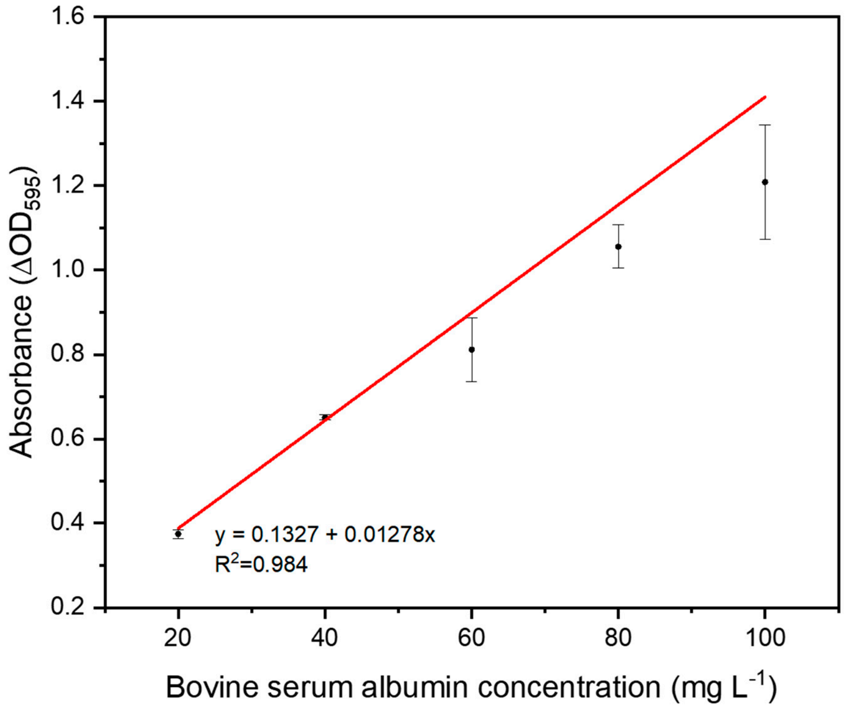

Figure S3. Standard curve of bovine serum protein. Values are means ± SD, n=3.

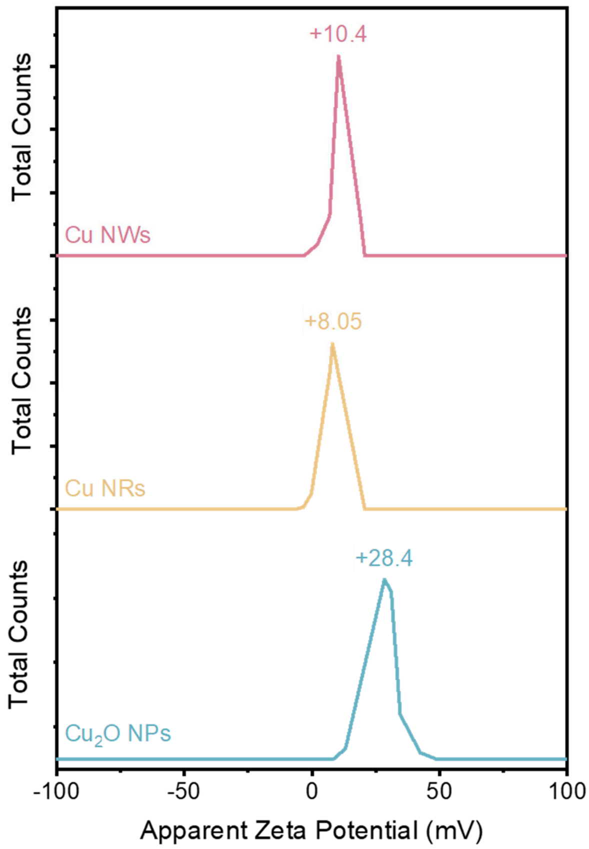

Figure S4. *Zeta* potential of Cu_2O NPs, Cu NRs and Cu NWs.

Table S1 Mass concentration (active ingredients) of Cu NM and control pesticide for mycelial growth test

Target	Concentration of mother liquor (mg L^{-1})	Final Concentration (mg L^{-1})					
		1	2	3	4	5	6
Cu_2O NPs	1000	50	60	70	80	90	100
Cu NRs	1000	20	30	40	50	60	70
Cu NWs	2000	75	100	125	150	175	200
$Cu(OH)_2$ (WP)	5000	100	200	250	300	400	500

Table S2 Mass concentration of Cu NMs for acute toxicity testing in adult zebrafish

Target	Exposure Concentration (mg L^{-1})					
	1	2	3	4	5	6
Cu_2O NPs	2.0	4.0	5.0	6.0	8.0	10.0
Cu NRs	0.2	0.4	0.5	0.6	0.8	1.0
Cu NWs	0.5	0.8	1.1	1.4	1.7	2.0

Table S3 The bioactivity of Cu NMs and Copper Hydroxide (WP) to *A. alternata*.

Target	Regression equation	EC_{50} （mg L^{-1}）	95% confidence limit （mg L^{-1}）	R^2	χ^2
Cu_2O NPs	y = -6.26+3.1x	104.24	96.64-126.52	0.936	2.361
Cu NRs	y = -5.4+2.77x	89.40	75.38-114.14	0.992	0.515
Cu NWs	y = -5.99+2.67x	166.88	157.45-202.19	0.978	1.143
$Cu(OH)_2$ (WP)	y = -3.07+1.36x	171.87	135.01-214.02	0.953	1.702
